# Novel targeted drugs for follicular and marginal zone lymphoma: a comprehensive review

**DOI:** 10.3389/fonc.2023.1170394

**Published:** 2023-05-03

**Authors:** Andrea Rivero, Pablo Mozas, Laura Magnano, Armando López-Guillermo

**Affiliations:** ^1^ Department of Hematology, Hospital Clínic de Barcelona, Barcelona, Spain; ^2^ Institut d’Investigacions Biomèdiques August Pi i Sunyer (IDIBAPS), Barcelona, Spain; ^3^ Universitat de Barcelona, Barcelona, Spain

**Keywords:** follicular lymphoma, marginal zone lymphoma, targeted therapy, immunotherapy, CAR-T cells

## Abstract

Although mostly incurable, indolent non-Hodgkin lymphomas (iNHL) are chronic diseases with a median overall survival approaching 20 years. In recent years, important advances in the knowledge of the biology of these lymphomas have led to the development of new drugs, mostly chemotherapy-free, with promising outcomes. With a median age of around 70 years at diagnosis, many patients with iNHL suffer from comorbid conditions that may limit treatment options. Therefore, nowadays, in the transition towards personalized medicine, several challenges lie ahead, such as identifying predictive markers for the selection of treatment, the adequate sequencing of available therapies, and the management of new and accumulated toxicities. In this review, we include a perspective on recent therapeutic advances in follicular and marginal zone lymphoma. We describe emerging data on approved and emerging novel therapies, such as targeted therapies (PI3K inhibitors, BTK inhibitors, EZH2 inhibitors), monoclonal antibodies and antibody-drug conjugates. Finally, we describe immune-directed approaches such as combinations with lenalidomide or the even more innovative bispecific T-cell engagers and chimeric antigen receptor T-cell therapy, which can achieve a high rate of durable responses with manageable toxicities, further obviating the need for chemotherapy.

## 1 Introduction

Indolent B-cell non-Hodgkin lymphomas (iNHL) include various lymphoproliferative disorders, being follicular lymphoma (FL) the most paradigmatic entity of this group of diseases. Marginal zone lymphoma (MZL), lymphoplasmacytic lymphoma/Waldenström macroglobulinemia (LPL/WM), and small lymphocytic lymphoma (SLL) are also part of this group. Overall, they comprise up to one third of all non-Hodgkin lymphomas (NHL). Since the median age at diagnosis ranges from 65 to 75 years (1), comorbidity is a factor to consider when selecting therapy (2). One of the main features of iNHL is their slow growing pattern and indolent behavior (3). With the introduction of rituximab in frontline therapy, most iNHL patients experience a long overall survival (OS), now approaching 20 years (4, 5). However, these diseases are considered incurable, and patients display a pattern of continuous relapses and remissions, and are exposed to the recurring need for treatment (6). In FL in particular, a progressively shorter duration of response (DoR) and progression-free survival (PFS) to subsequent treatments (second or later lines of therapy) has been well documented (7, 8).

Another attribute of iNHL is the risk of transformation to an aggressive lymphoma (HT), which occurs in around 1-3% of patients per year in FL (9–11) and in 1% per year in MZL patients (12). Both HT and early relapse within 2 years after initial immunochemotherapy (ICT, POD24) confer a poor prognosis when patients are treated with conventional therapies, highlighting an unmet need for patients with relapsed/refractory (RR) iNHL (13–16).

Despite the plethora of prognostic indexes available for indolent lymphoproliferative disorders (17–21), some refined with genetic data (22, 23), none of them are predictive of response to therapy and they cannot reliably identify the subgroup of patients with poor outcomes, which makes individual prognostication unfeasible at this time.

Current treatment of FL is commonly based on radiotherapy and rituximab (localized stages), watchful waiting (advanced-stage, low tumor burden patients), and ICT followed by rituximab maintenance (high tumor burden patients) (24). MZL is generally treated with immuno- or immunochemotherapy (25).

**Figure 1:**
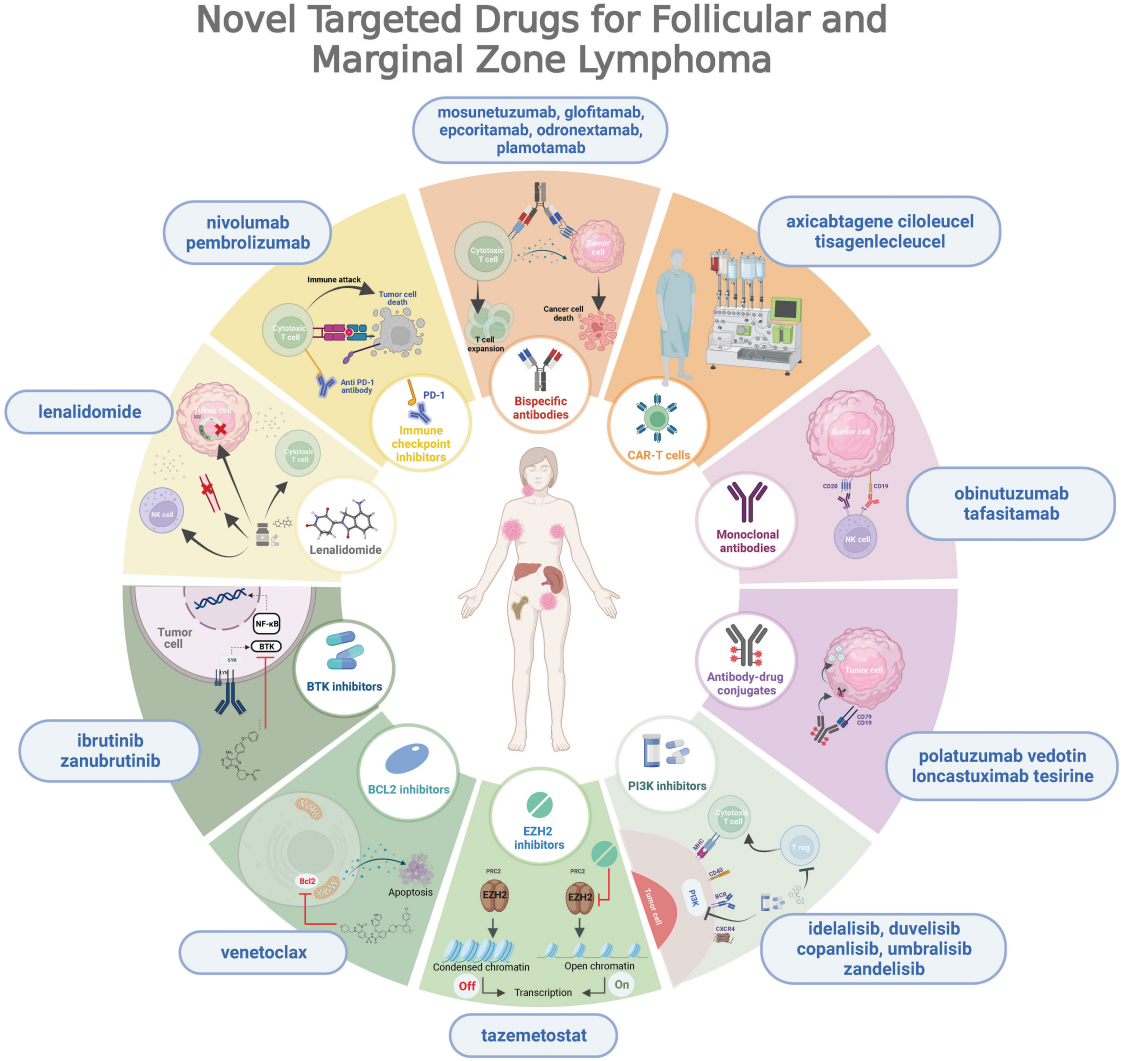
Overview of novel targeted drugs for follicular and marginal zone lymphoma, depicting their mechanisms of action.

In recent years, we have witnessed important advances in the knowledge of the biology of these lymphomas, which have facilitated the development of new drugs with promising results (26, 27) (**Figure 1**). The emergence of new therapies modulating proliferation pathways or the patient’s immunity to attack the tumor opens the door to a very interesting chemo-free therapeutic landscape.

Despite the availability of new drugs, the impact of these diseases on the quantity and quality of life of these patients continues to be significant. In fact, lymphoma-related mortality remains the most common cause of death (28, 29). On the other hand, managing new toxicities associated with novel therapies, as well as long-term and cumulative toxicities, represents a unique challenge.

Finally, an additional conundrum is the optimal sequencing of available therapies and choosing the treatment that best fits the profile of each patient. In this review, we collected published data on novel agents used for the treatment of FL and MZL.

## 2 Small molecules with targeted action

**Figure 2:**
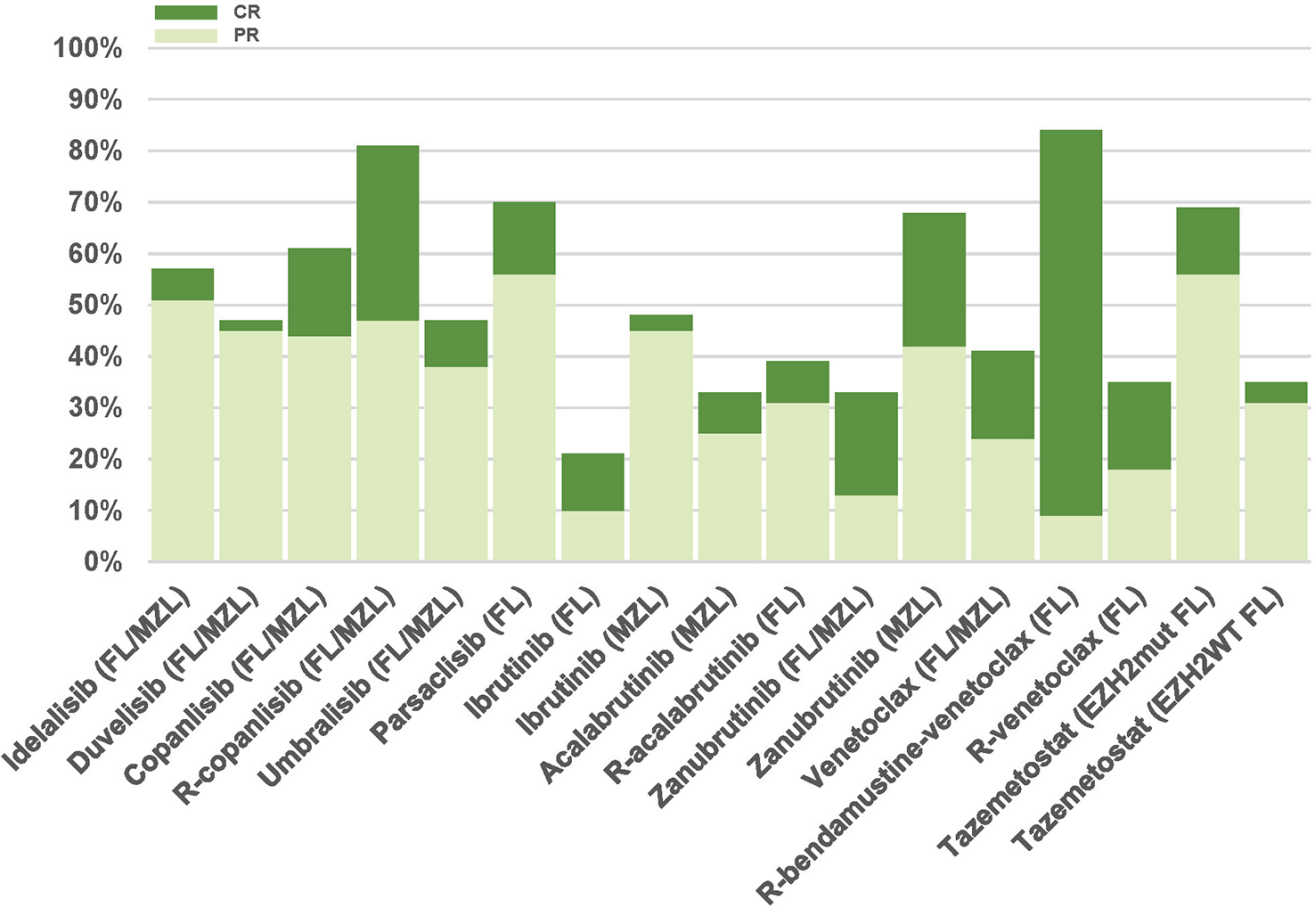
Partial response (light green) and complete response rates (dark green) in trials evaluating small molecules with targeted action for FL/MZL patients having received at least 2 prior lines of therapy. FL, follicular lymphoma; MZL, marginal zone lymphoma; R, rituximab; mut, mutated; WT, wild type.

Relevant clinical trials evaluating small molecules with targeted action are summarized in **Table 1**. **Figure 2** depicts response rates reported in clinical trials.

**Table 1:** Summary of clinical trials with small molecules with targeted action.

Drug	Mechanism of action	Type of study	Patients (n)		Median number of prior lines	ORR	CRR	DoR, median (months)	PFS	OS	Toxicities (grade ≥3)	Reference
			FL	MZL								
Idelalisib	PI3Ki	Phase 2 CT	72	15	4	57%	6%	12.5	47% at 1y (median PFS: 11 mo)	80% at 1y	Neutropenia: 27% Elevated liver enzymes: 13% Diarrhea: 13% Pneumonia: 7%	(30)
Duvelisib		Phase 2 CT	83	18	3	47%	2%	10	62% at 6-mo (Median PFS: 10 mo)	77% at 1y	Neutropenia: 25% Diarrhea: 15% Anemia: 15% Thrombocytopenia: 12%	DYNAMO (31)
Copanlisib		Phase 2 CT	104	23	3	61%	17%	14	34% at 2y (Median PFS: 13 mo)	Median, 43 69% at 2y	Hyperglycemia: 40% Diarrhea: 9% Hypertension: 24% Neutropenia: 24%	CHRONOS-1 (32)
Rituximab-copanlisib		Phase 3 CT	R-CO: 184 R: 91	R-CO: 66 R: 29	2	R-CO:81% R: 48%	R-CO: 3% R: 15	R-CO: 20 R: 17	Median PFS: R-CO: 22 R: 14	At 3y R-CO: 83% R: 81%	Hyperglycemia: 56% Hypertension: 40% Neutropenia: 16% Pneumonia: 6% Lymphopenia: 6% Diarrhea: 5%	CHRONOS-3 (33)
Umbralisib		Phase 2 CT	117	69	2	47%	9%	MZL: NR FL: 11	Median PFS: MZL: not reached FL: 11	NR	Neutropenia: 12% Diarrhea: 10% Elevated liver enzymes: 7%	UNITY-NHL (34)
Zandelisib ± rituximab		Phase 1b CT	63	4	2	NR	Neutropenia: 15% Diarrhea: 13% Pneumonia: 9%, ALT increase: 5% Colitis: 3%	35				
Parsaclisib		Phase 2 CT	106	–	2	70%	14%	NR	Median, 16	NR	Neutropenia: 9% Diarrhea: 6% Colitis: 4% Pleural effusion: 2%	CITADEL-203 (35)
Parsaclisib		Phase 2 CT	–	99	2	54%	Not reported	9	Median, 14	NR	Neutropenia: 13% Diarrhea: 5% Colitis: 3%	CITADEL-204 (36)
Ibrutinib	BTKi	Phase 2 CT	110	–	3	21%	11%	19	Median, 5	61% at 30 mo	Neutropenia: 14% Anemia: 9% Pneumonia: 6% Fatigue: 6% Diarrhea: 5%	DAWN (37)
Ibrutinib		Phase 2 CT	–	63	2	48%	3%	19	Median, 14	81% at 18 mo	Anemia: 14% Pneumonia: 8% Fatigue: 6% Cellulitis: 5% Diarrhea: 5% Hypertension: 5% Neutropenia: 5% Lymphopenia: 5%	(38)
Acalabrutinib ± rituximab		Phase 1b CT	TN: 13 R/R: 27	–	2 (R/R)	TN AR: 92% R/R AR: 39% R/R A: 33%	TN AR: 31% RR AR: 8% RR A: 8%	NR	NR	NR	Hypertension: 8% Elevated liver enzymes: 5% Cellulitis: 5%	(39)
Acalabrutinib		Phase 1b/2 CT	–	43	1	53%	13%	76% at 1y	Median, 27 67% at 1-y	91% at 1y	Neutropenia: 14% Anemia: 7% Dyspnea: 5% Thrombocytopenia: 5%	ACE-LY-003 (40)
Zanubrutinib		Phase 2 CT	–	68	2	68%	26%	93% at 1y	83% at 15 m	93% at 15 mo	Covid-19 pneumonia: 4% Pneumonia: 3% Diarrhea: 3% Pyrexia: 3%	MAGNOLIA (41)
Zanubrutinib		Phase 1/2 CT	33	20	MZL: 2 FL: 3	FL: 36% MZL: 80%	FL: 18% MZL: 20%	Median not reached	FL: - Median, 10 - 26% at 3y MZL: - Median, not reached - 72% at 3y	FL: - Median, not reached - 76% at 3y MZL: - Median, not reached - 84% at 3y	Bleeding: 4% Hypertension: ~5% SPM: ~5% Infections: ~25% Anemia: ~15% Neutropenia: ~19% Thrombocytopenia: ~5%	BGB-3111-AU-003 (42)
Rituximab-ibrutinib		Phase 2 CT	80	–	Frontline	~80%	~45%	Median not reached	~66% at 30 m	~98% at 30 mo	Fatigue: 10% Diarrhea: 6% Myalgia: 6% Rash: 6% Arthralgia: 5% Pyrexia: 6%	(43)
Venetoclax	BCL2i	Phase 1 CT	29	3	FL: 3 MZL: 4	41%	FL: 17% MZL: 0	FL: 27 MZL: 21	FL: - Median PFS 11 MZL - Median PFS 20	NR	Anemia: 11% Neutropenia: 13%	(44)
Venetoclax + R/O-CHOP		Phase 1b CT	10	4	FL: 4 MZL: 1	88%	78%	NR	Median, not reached ~95% at 1 y	NR	Neutropenia (~57%), febrile neutropenia (~30%), thrombocytopenia (~25%), anemia (~20%)	CAVALLI (45)
R- venetoclax ± bendamustina		Phase 2 CT	RV: 52 RVB: 51	–	3	RV: 35% RVB: 84%	RV: 17% RVB: 75%	NR	RV: - Median, 6	NR	RV: Neutropenia: 25% Thrombocytopenia: 8% anemia (6%). RVB: Neutropenia: 59% Thrombocytopenia: 45% Anemia: 10% Febrile neutropenia: 12% Vomiting: 10% Hypokalemia: 12%	CONTRALTO (46)
Tazemetostat	EZH2i	Phase 2 CT	99	–	*EZH2* ^mut^: 2 *EZH2* ^WT^: 3	*EZH2* ^mut^: 69% *EZH2* ^WT^: 35%	*EZH2* ^mut^: 13% *EZH2* ^WT^: 4%	*EZH2* ^mut^: 11 *EZH2* ^WT^: 13	Median PFS: *EZH2* ^mut^: 14 *EZH2* ^WT^: 11	NR	Thrombocytopenia: 3% Neutropenia: 3% Anemia: 2%	(47)
Tazemetostat-rituximab-lenalidomide		Phase 1b CT	44	–	≥2 (32%)	98%	51%	NR	NR	NR	Neutropenia: 34%	SYMPHONY-1 (48)

ORR, overall response rate; CRR, complete response rate; DoR, duration of response; R^2^, rituximab-lenalidomide; R, rituximab; O, obinutuzumab; CIT, chemoimmunotherapy; CT, clinical trial; R-CO, rituximab-copanlisib; TN, treatment naïve; R/R, relapsed/refractory; RV, rituximab-venetoclax; RVB, rituximab-venetoclax-bendamustine; A, acalabrutinib; NR, Not reported.

### 2.1 Phosphoinositide 3-kinase (PI3K) inhibitors

Since PI3K is an integral part of B-cell receptor (BCR) signaling, the rationale behind the use of PI3K inhibitors (PI3Ki) in clinical practice is solid. However, their widespread use has been limited by toxicity, namely immune-related adverse events and opportunistic infections, which make the use of antiviral and *Pneumocystis jirovecii* prophylaxis mandatory.

Idelalisib, an orally active selective PI3Kδ inhibitor, was the first PI3Ki to be approved (30). The pivotal study included 72 FL and 15 MZL patients, with a median of 4 prior lines of treatment. With an overall response ratio (ORR) of 57% (only 6% complete response; CR), the median PFS was 11 months (mo). Grade ≥3 (G ≥3) adverse events (AEs) included diarrhea (13%), increased alanine aminotransferase (ALT) (13%) and neutropenia (27%).

Duvelisib, an oral PI3Kδ/γ inhibitor, was tested in the phase 2 DYNAMO study (31), including 129 patients with R/R FL and a median of 3 previous lines of therapy. It demonstrated an ORR of 47% (CR 2%) and a median PFS of 9.5 mo. The rate of G ≥3 AEs was fairly comparable to that of its predecessor.

Copanlisib is an intravenous PI3Kδ/α and CK1ϵ inhibitor. In the CHRONOS-1 study (32), this drug was tested in 142 R/R FL patients with a median of 3 prior therapies. Although efficacy was in line with that of other PI3Ki, the toxicity profile was different, with a high rate of G ≥3 infusion related hypertension (40%) and hyperglycemia (24%). Other relevant G ≥3 AEs were diarrhea, colitis, elevated liver enzymes and pneumonitis.

Umbralisib, a PI3Kδ and CK1ϵ inhibitor that is delivered orally, was tested in the phase 2 UNITY-NHL study (34), with 117 FL and 69 MZL patients R/R to a median of two lines of treatment. For FL patients, the ORR and CR were 45% and 5%, respectively, with a median PFS of 10.6 mo. ORR and CR for MZL patients was 49% and 16%, respectively, and the median PFS was not reached. The toxicity profile that was initially described for this drug was similar to that of other members of the family. Based on this trial, umbralisib was FDA-approved in 2021 for MZL that had failed 3 prior lines of therapy. However, recently emerging safety concerns have led to its withdrawal (49).

Zandelisib, a potent PI3Kδ inhibitor, has been tested in R/R FL and CLL in a dose-escalation, dose-expansion phase 1b trial (50). The most common G ≥3 AEs were neutropenia, diarrhea, pneumonia, ALT increase, and colitis. The selected therapeutic dose was 60 mg once daily on an intermittent dosing schedule (daily for two 28-day cycles and on days 1-7 thereafter). Efficacy data from phase 2 and 3 studies are awaited.

Two phase 2 studies have evaluated the oral PI3Kδ inhibitor parsaclisib given daily as a high-dose 8-week induction, followed by maintenance with either higher weekly or lower daily doses. Reduced daily dosing was considered the preferred approach, and crossover from the higher weekly dose was allowed. In 106 R/R FL patients with a median of 2 prior lines of therapy (CITADEL-203 (35)), the ORR was 70% (14% CR) and median PFS was 16 mo. In 99 BTKi-naïve R/R MZL patients (CITADEL-204 (36)), ORR was 54% (CR 6%) and the median PFS was 19 mo. Apart from a markedly lower rate of hepatic toxicity, the AE profile was comparable to that of other PI3Kδ inhibitors.

Although the combinations of PI3Ki with chemotherapy have not been successful due to increased toxicity, associations with rituximab have shown promise. In the phase 3 CHRONOS-3 study (33), R-copanlisib was evaluated against R-placebo in 458 non-rituximab-refractory, R/R iNHL patients. Median PFS was 22 mo for R-copanlisib, vs. 13.8 for R-placebo, without safety alerts. One caveat of the study is that it does not answer the question whether rituximab adds any benefit to copanlisib alone.

Based on these results, PI3Ki are generally considered to be active although with substantial toxicity, including hepatotoxicity, colitis, pneumonitis and infection, with high levels of discontinuation across studies (15–30%). In this sense, intermittent dosing schedules have been developed, with the intention of improving tolerance and long-term adherence. Considering the aforementioned data, the role of PI3K inhibitors in B-NHL remains unclear.

### 2.2 Bruton tyrosine kinase (BTK) inhibitors

Although BTK is central to BCR signaling and an important target in other B-cell malignancies such as chronic lymphocytic leukemia (CLL), mantle cell lymphoma or WM, results of the phase 2 DAWN study (37) (ibrutinib in R/R FL) were disappointing, with an ORR of only 20%.

Ibrutinib is significantly more active in MZL (38). In a phase 2 study including 63 patients with rituximab-exposed R/R MZL (MALT, 51%; nodal, 27%; and splenic, 22%), the ORR was 48% (CR 3%) and the median PFS was 14 mo, with the most common G ≥3 AEs being anemia, pneumonia and fatigue, with a 10% discontinuation rate. Ibrutinib was FDA-approved for the management of patients with R/R MZL, based on results from this trial. Recently, long term follow-up was reported (51). After a median follow-up of 33 months, ORR was 58%, consistent across all subtypes. Median DoR and PFS were 28 and 16 months, respectively. Median OS was not reached. Mutations in *KMT2D* and *CARD11* were associated with an inferior outcome compared to *MYD88*-mutated cases.

The MALIBU-IELSG47 study assessing ibrutinib-rituximab in untreated MZL is ongoing (NCT03697512), with the intention of establishing whether the addition of an anti-CD20 monoclonal antibody (MoAb) might improve outcomes.

Zanubrutinib is a second-generation oral covalent (irreversible) BTKi that has been explored in CLL and MZL. The phase 2 MAGNOLIA study (41) evaluated zanubrutinib 160 mg orally bid in 68 R/R MZL patients who had received at least 1 prior line of therapy including an anti-CD20 MoAb (median of prior lines of therapy: 2). The ORR was 68% (CR 26%), the median PFS had not been reached with the reported follow-up, and the overall toxicity profile was slightly better than that of ibrutinib. These data granted zanubrutinib FDA approval for R/R MZL which, together with ibrutinib, are interesting non-chemotherapy strategies for this group of patients. In a later phase 1/2 trial (42), zanubrutinib confirmed its efficacy for in R/R MZL and showed slightly lower activity in R/R FL (ORR 36%, CR 18%, median PFS 10 months). Preliminary studies (39, 40) have explored the therapeutic potential of acalabrutinib with or without rituximab, but data are still immature.

Furthermore, anti-CD20/BTKi combinations might also be efficacious. Importantly, a phase 2 study (43) evaluated the rituximab-ibrutinib combination in frontline FL. ORR was around 80% (CR 40-50%) and 30-month PFS and OS were ~66 and ~98%, respectively, with the most common AEs being fatigue, diarrhea and nausea. These very favorable results must be examined under the consideration that the population included in the study was highly treatment-sensitive, and results of the phase 3 PERSPECTIVE trial (NCT02947347) are yet to be published.

### 2.3 BCL2 inhibitors

BCL2 is an anti-apoptotic protein that provides survival advantage for malignant B cells and is involved in tumorigenesis, disease progression and chemoresistance (52). Despite the characteristic *BCL2::IGH* rearrangement and BCL2 overexpression in FL (53), results of BCL2 inhibitors (BCL2i) in iNHL have been somewhat underwhelming. Long-term follow-up of the phase 1 study evaluating single-agent venetoclax in R/R iNHL (44) showed a median PFS of 11 and 21 mo for FL and MZL, respectively. ORR (CR) were 38% (17%) and 67% (0%) for FL and MZL patients, respectively. Hematological toxicity was moderate (around 20% for neutropenia, anemia and thrombocytopenia, all grades) and other frequent AEs included nausea, diarrhea and fatigue.

Regarding CIT-BCL2i associations, the phase 1b CAVALLI trial (45) evaluated venetoclax combined with R-CHOP or G-CHOP in frontline or R/R NHL (43% FL). Cytopenias were significant AEs, particularly in the obinutuzumab arm, which led to the selection of a higher, time-limited venetoclax dosing (800 mg days 4-10 of cycle 1 and days 1-10 thereafter). Further exploration of these combinations is under way. In turn, the rituximab-bendamustine-venetoclax combination was compared to rituximab-bendamustine in the phase 2 CONTRALTO trial (46), evaluating 163 R/R FL patients. Although there was a tendency towards a higher efficacy in the venetoclax-containing arm, a higher frequency of hematologic toxicity resulted in more reduced dosing and treatment discontinuation, which makes the combination unlikely to reach clinical practice.

In the frontline setting, the phase 2 PrECOG-0403 trial examined the combination of venetoclax, bendamustine and obinutuzumab in patients with high tumor burden FL (n = 56) (54). Outcomes were favorable, with a CR of 73%, a 2-year PFS of 86%, and a 2-year OS of 94%. However, toxicities were a concern, with a high frequency of G ≥3 AEs including tumor lysis syndrome (14%), thrombocytopenia (14%), neutropenia (16%) and opportunistic infections such as CMV encephalitis, which suggests the high immunosuppressive potential of this regimen.

Obinutuzumab-venetoclax is also being studied in combination with lenalidomide in the frontline treatment of advanced FL in the phase 1/2 LEVERAGE study (NCT03980171).

### 2.4 Epigenetic therapies: *EZH2* inhibitors

The pathogenesis of FL is highly dependent on the disruption of epigenetic regulators (55), of which the enhancer of zeste homolog 2 (EZH2) is crucial for germinal center biology. It catalyzes methylation of H3K27, resulting in controlled repression of gene transcription, critical for regulating genes involved in the cell cycle, B-cell differentiation and maturation. Activating *EZH2* mutations are considered an early lymphomagenic event and are present in around one in five FL cases. Tazemetostat, an oral *EZH2* inhibitor (EZH2i), was the first in the group and is active against both mutant (EZH2^mut^) and wild-type (EZH2^wt^) EZH2. The pivotal phase 2 study (47) evaluated the activity and safety of twice-daily tazemetostat in 99 R/R FL patients (including grade 3B and transformed FL), until progression or unacceptable toxicity. The ORR (CR) was 35% (4%) and 69% (2%) for wild-type and mutated *EZH2* patients, respectively. With a median follow-up of 36 and 22 months for the wild-type and mutated cohorts, the median DoR was 13 and 11 mo, respectively. Toxicity was acceptable with fatigue as the most common non-hematological AE. Based on this trial, accelerated FDA approval was given for R/R FL (failing ≥2 prior lines if *EZH2* ^mut^ or if no satisfactory alternative treatment is available).

Tazemetostat has also been combined with R (2) in the phase 1b SYMPHONY-1 study (48), with favorable efficacy and no new toxicity concerns. The phase 3 portion of the study will expand on these data in over 500 patients. The phase 2 SYMPHONY-2 trial will examine tazemetostat in combination with rituximab in R/R FL patients (NCT04762160). Indeed, a study combining atezolizumab with obinutuzumab has completed recruitment (NCT02220842), results are awaited.

Tazemetostat-lenalidomide-obinutuzumab was studied in R/R FL in a phase 1b/2 study (56) with promising activity (ORR 78%, CR 72% and 36-month PFS 68%) but unacceptable toxicities.

## 3 Harnessing the immune system

**Figure 3:**
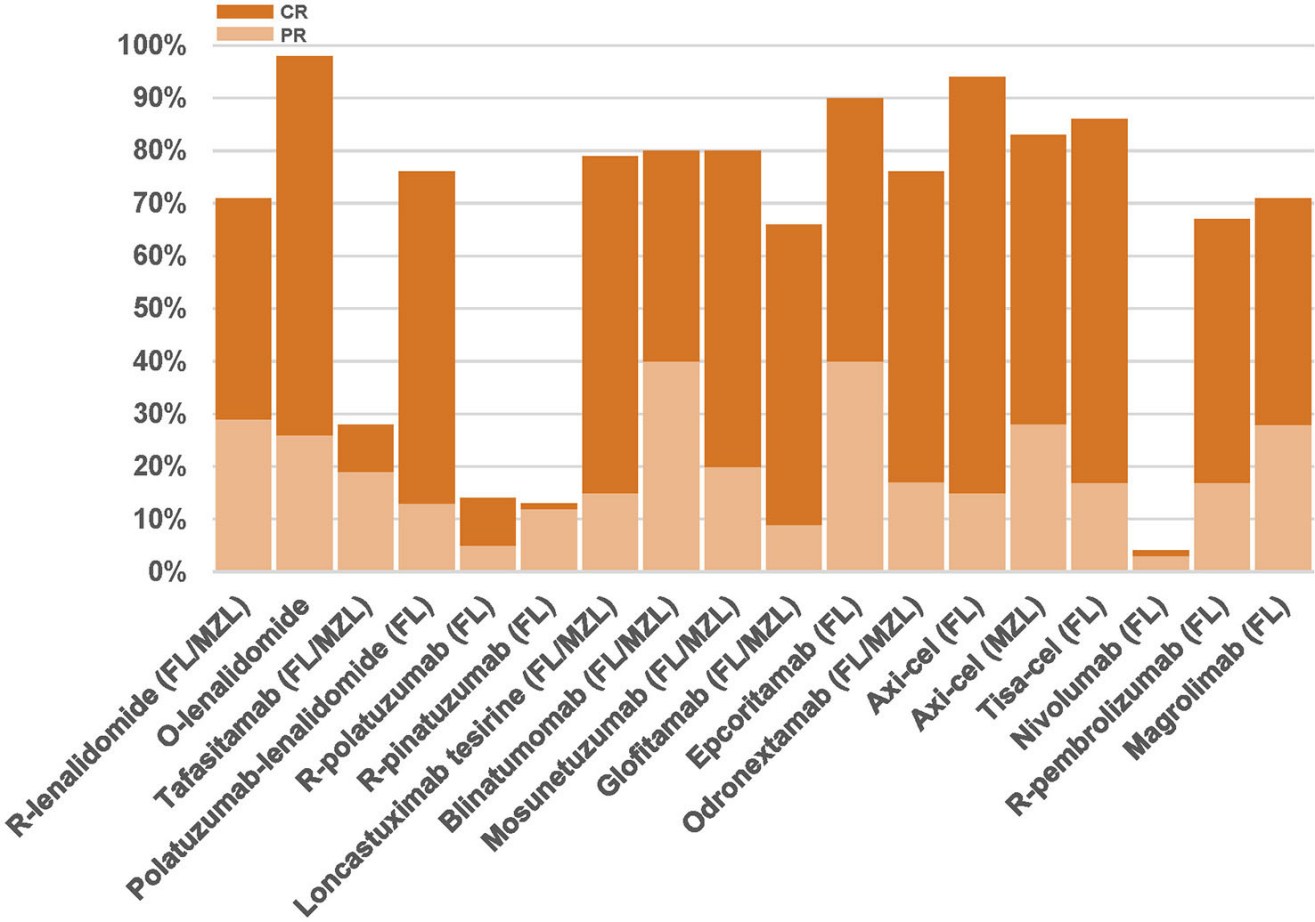
Partial response (light orange) and complete response rates (dark orange) in trials evaluating immune-directed therapies for FL/MZL patients having received at least 2 prior lines of therapy. FL, follicular lymphoma; MZL, marginal zone lymphoma; R, rituximab; O, obinutuzumab.

Relevant clinical trials evaluating immunotherapies are summarized in **Table 2**. **Figure 3** depicts response rates reported in clinical trials. **Table 3** is a snapshot of G ≥3 toxicities occurring in ≥10% of patients in iNHL patients treated with novel drugs.

**Table 2:** Summary of clinical trials with immunotherapies.

Drug	Mechanism of action	Type of study	Patients (n)		Median number of prior lines	ORR	CRR	DoR, median (months)	PFS	OS	Toxicities (grade ≥3)	Reference
			FL	MZL								
R^2^	Immunomodulator	Phase 3 CT	R-chemo: 517 R^2^: 513	–	Frontline	R-CT: 65% R^2^: 61%	R-CT: 53% R^2^: 48%	NA	At 6y R-CT: 59% R^2^: 60%	89% at 6y (both arms)	Neutropenia: 32% Skin reactions: 7%	RELEVANCE (57)
R^2^		Phase 3 CT	R: 148 R^2^: 147	R: 32 R^2^: 31	1	R: 53% R^2^: 78%	R: 18% R^2^: 34%	R: 22, R^2^: 37	At 2y R: 36% R^2^: 58%	At 2y R: 87% R^2^: 93%	Neutropenia: 50% Diarrhea: 31% (all grades) Anemia: 5%	AUGMENT (58)
R^2^		Phase 3b CT (induction phase data)	318	76	2	71%	42%	NR	Median, 51	NR	Neutropenia: 37% Thrombocytopenia: 6% Anemia: 5% Fatigue: 5%	MAGNIFY (59)
Obinutuzumab-lenalidomide		Phase 2 CT	90	–	Frontline	98%	92%	NR	96% at 2y	NR	Neutropenia: 16% Skin reactions: 10%	(60)
Obinutuzumab-lenalidomide		Phase 1/2 CT	57	4	2	98%	72%	NR	73% at 2y	NR	Neutropenia: 17% Thrombocytopenia: 11% Fatigue: 8% Skin reaction: 6% Cough: 5%	(61)
Tafasitamab	Anti-CD19 monoclonal antibody	Phase 2 CT	34	9	3	FL: 29%, MZL: 27%	FL: 9%, MZL: 18%	FL: 26, MZL: 7	NR	NR	Infuse reactions: 3% Neutropenia: 17%	(62)
Polatuzumab + lenalidomide	ADC	Phase 1/2 CT	46	–	3	76%	63%	NR	NR	NR	Neutropenia: 55% Thrombocytopenia: 25% Anemia: 14% Infections: 25% Diarrhea: 4%	(63)
R-polatuzumab vs. R-pinatuzumab		Phase 2 CT	R-PO: 20 R-PI: 21	–	2 3	14% 13%	9% 1%	9 7	15 13	NR NR	R-polatuzumab arm: Neutropenia: 23% Anemia: 8% Peripheral neuropathy 11%	(64)
Loncastuximab tesirine		Phase 1 CT	14	6	3	79%	64%	5	NR	NR	Thrombocytopenia: 26% Neutropenia: 40% Anemia: 15% GGT increased: 21%	(65)
Blinatumomab	CD3xCD19 bispecific antibody	Phase 1 CT	28	2	3	80%	40%	24	NR	NR	Neurotoxicity: 22%, Lymphopenia: 79% Thrombocytopenia: 12% Neutropenia: 17% Hyperglycemia: 12% Anemia: 7% Pyrexia: 4%	(66)
Mosunetuzumab	CD20xCD3 bispecific antibody	Phase 1 CT	65	2	3	45%	33%	17	NR	NR	CRS: 1% Neutropenia: 2.5% Hypophosphatemia: 15% Pneumonia 2.5%	(67)
Mosunetuzumab		Phase 2 CT	90	–	3	80%	60%	23	21	NR	CRS: 2.2% ICANS: 0% Neutropenia: 25% Hypophosphatemia:17% Anemia: 8% Thrombocytopenia: 4%	GO29781 (68)
Glofitamab		Phase 1 CT	44	1	3	71%	48%	NR	12	NR	CRS: 3.5% ICANS: 1% Neutropenia: 25% Anemia: 8% Pneumonia: 3%	(69)
Epcoritamab		Phase 1/2 CT	11	–	5	90%	50%	NR	NR	NR	Anemia: 13% Pyrexia: 6% Hypotension: 6%	(70)
Epcoritamab + R^2^		Phase 2 CT	36	–	0 (First line)	94%	86%	–	–	–	CRS: 0% Neutropenia: 24% Headache: 2% Rash: 7%	(71)
Odronextamab		Phase 1 CT	40	6	3	FL: 78%, MZL: 67%	FL: 63%, MZL: 33%	FL: 13, MZL: 18	NR	NR	CRS: 7% Neutropenia: 19% Anemia: 25% Thrombocytopenia: 14% Hypophosphatemia: 19%	(72)
Odronextamab		Phase 2 T	96	–	3	81%	75%	18	20	NR	COVID-19: 5%	ELM-2 (73)
CTL019 cells	Anti-CD19 CAR-T cells (4-1BB)	Case-series study	14	–	5	89%	71%	NR	70% at 28 mo	93% at 28 mo	CRS: 18%* ICANS: 11% Cytopenias: NR Infection: 29%	(74, 75)
AntiCD19 CAR-T (Seattle)	Anti-CD19 CAR-T cells (4-1BB)	Phase 1/2 CT	21 FL:8 tFL: 13	–	FL: 4 tFL: 5	FL: 88% tFL: 46%	FL: 88% tFL: 46%	FL: 24 tFL: 10	FL: 80% at 18 mo tFL: 20% at 18 mo	FL: 100% at 18 mo tFL: 45% at 18 mo	CRS: 0 ICANS: 0 Cytopenias: NR Infection: NR	(76)
Axi-cel	Anti-CD19 CAR-T cells (CD28)	Phase 2 CT	124	22	3	FL: 94% MZL: 83%	FL: 79% MZL: 55%	FL: Not reached MZL: 11	65% at 18 mo	87% at 18 mo	CRS: 7%** ICANS: 19% Cytopenias: 70% Infection: 18%	ZUMA 5 (77)
Tisa-cel	Anti-CD19 CAR-T cells (4-1BB)	Phase 2 CT	97	–	4	86%	69%	65% at 24 mo	57% at 24 mo	88% at 24 mo	CRS: 0** ICANS: 3%^+^ Cytopenias: 32% Infection: 5%	ELARA (78)
Nivolumab	Immune checkpoint inhibitor	Phase 2 CT	92	–	3	4%	1%	19	2	NR	Diarrhea: 2% Neutropenia: 2% Anemia: 1%	(79)
Pembrolizumab + Rituximab		Phase 2 CT	30	–	2	67%	50%	18	13	NR	Diarrhea: 3% Pancreatitis: 3% AST increased: 3% ALT increased: 3%	(80)
Magrolimab	Anti-CD47	Phase 1 CT	7	–	4	71%	43%	NR	NR	NR	All grades: Fatigue: ~60% Diarrhea: ~50% Anemia: ~40% Infusion-related reaction: ~40% Neutropenia: ~30% Insomnia: ~30%	(81)

ORR, overall response rate; CRR, complete response rate; DoR, duration of response; NR, not reported; tFL, transformed follicular lymphoma; ADC, antibody-drug conjugate; R, rituximab; R-PO, rituximab-polatuzumab; R-PI, rituximab-pinatuzumab; NA, Not available.

*Penn scale; **Lee scale.

^+^Neurological events were graded according to CTCAE v.4.03 and American Society of Transplantation and Cellular Therapy ICANS consensus grading.

**Table 3:** Grade 3 or higher adverse events occurring in 10% or more of the patients.

	Drug (entity)	Neutropenia	Anemia	Thrombocytopenia	Lymphopenia	Infections	Neutropenic fever	Skin reactions	Diarrhea/colitis	Fatigue	Peripheral neuropathy	Vomiting	Hyperglycemia	Arterial hypertension	Elevated liver enzymes	Hypokalemia	Hypophosphatemia	CRS	ICANS/neurotoxicity
PI3Ki	Idelalisib (FL/MZL)	•							•						•				
	Duvelisib (FL/MZL)	•	•	•					•										
	Copanlisib (FL/MZL)	•											•	•					
	R-copanlisib (FL/MZL)	•											•	•					
	Umbralisib (FL/MZL)	•							•										
	Zandelisib ± R (FL/MZL)	•							•										
	Parsaclisib (FL)	•																	
BTKi	Ibrutinib (FL)	•																	
	Ibrutinib (MZL)		•																
	R-ibrutinib (FL)									•									
	Acalabrutinib (MZL)	•																	
	R-acalabrutinib (FL)																		
	Zanubrutinib (FL/MZL)	•	•			•													
	Zanubrutinib (MZL)																		
BCL2i	Venetoclax (FL/MZL)	•	•																
	R-bendamustine-venetoclax (FL)	•	•	•			•					•				•			
	R-venetoclax (FL)	•																	
EZH2i	Tazemetostat (FL)																		
	Tazemetostat-R-lenalidomide (FL)	•																	
Immunomodulator	R-lenalidomide (FL/MZL)	•																	
	O-lenalidomide	•		•				•											
Anti-CD19 monoclonal antibody	Tafasitamab (FL/MZL)	•																	
Antibody-drug conjugates	Polatuzumab-lenalidomide (FL)	•	•	•		•													
	R-polatuzumab (FL)	•									•								
	Loncastuximab tesirine (FL/MZL)	•	•	•											•				
Bispecific antibodies	Blinatumomab (FL/MZL)	•		•	•								•						•
	Mosunetuzumab (FL/MZL)	•															•		
	Glofitamab (FL/MZL)	•																	
	Epcoritamab (FL)		•																
	Odronextamab (FL/MZL)	•	•	•													•		
CAR-T cells	Axi-cel (FL/MZL)	•	•			•												•	
	Tisa-cel (FL)	•	•				•												
Immune checkpoint inhibitor	Nivolumab (FL)																		
	R-pembrolizumab (FL)																		

Rows with no dots indicate that the corresponding drug did not show any of those G ≥3 AEs in ≥10% of patients. FL, follicular lymphoma; MZL, marginal zone lymphoma; CRS, cytokine release syndrome; ICANS, immune effector cell-associated neurotoxicity syndrome; R, rituximab; O, obinutuzumab.

### 3.1 Immunomodulators: lenalidomide

This small molecule has immunomodulatory properties that derive from several mechanisms, namely the inhibitory effects of the E3 ubiquitin ligase complex, in which cereblon is involved, resulting in the degradation of multiple transcription factors and consequent direct apoptotic death (82). It also exerts direct cytotoxicity, antibody dependent cellular cytotoxicity mediated by T- and NK-cell stimulation, as well as cytotoxic T-cell activity, reduction in regulatory T-cells, resensitization to anti-CD20 antibodies, and synergies with other BCR targeting agents. Moreover, the modulation of inflammatory cytokine production and checkpoint inhibitor expression can disrupt the protective tumor microenvironment.

Initially, single-agent activity of lenalidomide was demonstrated in 43 patients with R/R iNHL (22 FL). In FL patients, the ORR was 27% (CR 9%) with a median PFS for all patients of 4 months. AEs, including rash, fatigue and neutropenia were predictable and manageable (83).

This agent was then studied in association with rituximab (R (2)) in iNHL both in the frontline and R/R settings. The initial phase 2 study of frontline R (2) demonstrated promising response rates in 50 FL (ORR: 98%, CR: 87%) and 30 MZL patients (ORR: 90%, CR: 67%) (84). These encouraging results were confirmed in the phase 3 RELEVANCE study (57), in which R (2) was randomized against ICT and maintenance in 1030 patients with untreated FL. Although superiority compared to ICT was not demonstrated (CR at 120 weeks: 48% for R (2) vs 53% for ICT), efficacy was comparable (3-y PFS 77 for R (2) vs 78% for ICT; 3-y OS: 94% for both arms). This led to the relevant affirmation that chemotherapy-free approaches can have comparable efficacy to that of ICT in FL. Concerning toxicity, there were some differences: ICT produced more G ≥3 neutropenia, any grade neutropenic fever, nausea, diarrhea and neuropathy, while G ≥3 cutaneous toxicity, any grade myalgia and muscle spasms were more frequent in the R (2) arm. This regimen can thus be an option for patients at risk of developing complications related to, or unwilling to suffer from some of the effects of, chemotherapy.

The early incorporation of immunomodulatory agents in the treatment strategy of iNHL might disrupt the protective microenvironment, improve the response to subsequent lines of therapy and potentially change the natural history of the disease.

In the R/R setting, the phase 3 AUGMENT trial (58) evaluated R (2) vs R in 295 patients with FL and 63 with MZL. All patients had previously received ≥2 doses of rituximab but none were rituximab-refractory. In a recent update of the study (median follow-up: 5.5 y) (85), median PFS for the R (2) arm was 28 mo, and 5-y OS was 83% and 77% for the R (2) and R arms, respectively. There was a confirmation of low rates of second primary malignancies and HT in both arms, and PFS, time to next treatment and OS favored the use of R (2), despite a higher rate of adverse events, namely neutropenia and infections. This study has been criticized for the choice of the comparator arm and the different duration of therapy between arms. Nevertheless, it resulted in the FDA approval of the R (2) regimen for R/R FL. The phase 3 MAGNIFY study, designed to ascertain the optimum duration of the R (2) regimen for R/R iNHL, included 318 FL patients. The interim analysis for all enrolled patients showed an ORR of 72% (42% CR) and a median PFS of 51 months in the FL subgroup; no unexpected AEs were encountered (86).

The combination of lenalidomide and obinutuzumab has also demonstrated favorable results for treatment-naïve FL (60) and R/R iNHL (61) patients, although its regulatory status lags behind that of R (2).

### 3.2 Tafasitamab, an anti-CD19 antibody

CD19 is broadly and homogeneously expressed throughout B-cell differentiation, up to the stage of plasma cell, which makes it an interesting target in the treatment of B-cell lymphomas (62, 87). Tafasitamab is a novel humanized, Fc-engineered, CD19 monoclonal antibody that induces an antibody-dependent cell-mediated and antigen-dependent cell-mediated phagocytosis (62, 88).

Tafasitamab has been investigated in a phase 2 trial (62) with 92 B-NHL patients (34 FL and 9 MZL). It was administered as an intravenous infusion of 12 mg/kg. The ORR was 29% and 27% for FL and MZL, respectively, and a CR was seen only in 9% of the FL patients. Its safety profile was acceptable, with a 12% of any-grade infusion-related reactions and 12% of neutropenia, few of them G ≥3.

A phase 3 trial is recruiting R/R FL or MZL patients to evaluate efficacy and safety of tafasitamab plus lenalidomide and rituximab (R2) compared to placebo plus R (2) (NCT04680052) (89). The IELSG49 study (NCT04646395) is an ongoing pilot study of tafasitamab and acalabrutinib in R/R MZL.

### 3.3 Antibody-drug conjugates

Antibody-drug conjugates (ADC) are agents that allow, through the monoclonal antibody as a carrier, for the targeted delivery of a cytotoxic small molecule that is internalized into the malignant cell (90, 91). Target antigens should be highly expressed on malignant cells with minimal expression elsewhere, reducing the risk for systemic toxicity (92).

These combined drugs targeting B-cell antigens have been developed in the spectrum of B-cell lymphomas, and two ADC are already approved for DLBCL patients: polatuzumab vedotin and loncastuximab tesirine (93, 94).

Polatuzumab vedotin is an anti-CD79b antibody conjugated with the microtubule inhibitor monomethyl auristatin E (MMAE). Polatuzumab vedotin has shown promising response rates alone or in combination with rituximab in patients with FL (ORR 70%, CR 45%) (64), with an acceptable safety profile but concerns about peripheral neuropathy. Although pinatuzumab vedotin (anti-CD22 antibody and MMAE) showed encouraging activity with an acceptable safety profile in R/R B-cell NHL (95), the R-polatuzumab combination seems to induce more durable responses (64).

Polatuzumab vedotin has been tested in combination with obinutuzumab and lenalidomide in a multicenter study including 56 R/R FL patients, showing a high CRR (76%) (63). Several combinations with this anti-CD79b antibody are under evaluation in various trials (NCT02729896, NCT03671018).

Loncastuximab tesirine is a novel humanized CD19-targeted ADC, which delivers a pyrrolobenzodiazepine dimer as a cytotoxic molecule (88, 91). Promising results have been shown in patients with R/R DLBCL as a single agent and combined with other drugs (88). Experience in FL was collected in a phase 1 trial enrolling 183 B-NHL patients, 14 of which were R/R FL patients and six were MZL. Overall, the ORR was 79% and the CRR was 64%, with a median DoR of 5 mo (65). Analyses on iNHL specifically are not yet available. In the field of R/R FL, loncastuximab is under investigation in clinical trials in combination with rituximab (NCT04998669) and with idelalisib (NCT04699461).

### 3.4 Bispecific antibodies

These novel agents represent a new way of targeting B cells. Bispecific antibodies are T-cell engagers that bind surface CD3 to recruit the patient’s T-cells and simultaneously bind to B domains like CD19, or most commonly in lymphoma therapy, CD20, triggering T-cell-mediated cytotoxicity against B cells (88, 91, 96). The role of these agents is gaining more interest due to the lack of special logistic needs in the genetic modification of T-cells or product manufacturing compared with CAR-T cells, becoming an “off-the-shelf” therapeutic tool.

The array of adverse events related to these agents is comparable to that found with CAR-T cell therapy, including cytokine release syndrome (CRS) and neurological side effects (ICANS). However, these adverse events are usually observed during the initial infusions and most of them are reversible, with a frequency and severity that are much lower than those observed with CAR T-cell products. Cytopenias, mainly neutropenia, usually develop, being mild and with recovery in most cases (90–92, 97).

Blinatumomab is a continuous intravenous infusion CD19xCD3 bispecific T-cell engager that has been studied in a phase I study in B-NHL including 28 R/R FL. The ORR was 80% (40% of them CR) with 50% of responder patients showing responses that lasted 2 years or longer. AEs included CRS in 75% of patients and G ≥3 neurotoxicity in 22% of patients (66). Its continuous intravenous administration for weeks and its toxicity profile limit the use of this bispecific antibody in B-cell NHL.

A combination of blinatumomab with lenalidomide in 18 heavily pretreated R/R B-NHL patients showed an ORR of 91%, a CRR of 55% and a median PFS of 8 mo; G ≥3 neurologic toxicity was documented in 28% of patients. Although the cases included in this study were predominantly DLBCL (n=7), three patients had FL and one MZL (98).

Mosunetuzumab is an intravenous CD20xCD3 bispecific antibody that has been evaluated in several phase 1 and 2 studies including patients with R/R B-NHL, showing promising results in the form of durable CR. One of the first phase 1 trials included 68 patients with iNHL (65 FL, 2 MZL and 1 SLL). The ORR, CR and DoR were 45%, 33% and 17 mo, respectively. The most frequently described adverse events were neutropenia (28%) and CRS (27%), the latter being grade G ≥3 in 1%. No severe (grade 4-5) neurologic AE were reported (67).

A phase 2 study evaluated the administration of mosunetuzumab in 90 patients with R/R FL and reported an ORR of 80% (60% CR), with a median DoR of 23 mo. Mosunetuzumab was administered in 21-day cycles with a step-up dose procedure. Patients who reached a CR were given 8 cycles of treatment, while patients who reached a partial response or had stable disease after cycle 8 continued treatment for up to 17 cycles. CRS was the most common adverse event in 44% of patients, predominantly grades 1 or 2 (26% and 17%, respectively). Neurologic events were also described in very low rates (5%) and all resolved. All-grade neutropenia was reported in 28% of patients (67). These data granted the positive opinion from the European Medicines Agency for FL patients R/R to ≥2 lines of treatment. In a recent update of this trial (68), available baseline biopsy samples were analyzed by means of whole exome sequencing, and response was shown to be independent of the mutational status of *EZH2*, *TP53*, *BCL2*, *CREBBP* and *KMT2D*.

Glofitamab is a dual CD20xCD3 bispecific engager with a 2:1 configuration that allows for bivalent binding to CD20 on B-cells, while maintaining monovalency for CD3 on T cells (69, 97). A phase I trial evaluated the drug in 171 patients with R/R B-NHL (44 with FL and only one patient with MZL). Seven days prior to the first dose of glofitamab, all patients received 1000 mg of obinutuzumab as a way to debulky the B-cell component, and then a step-up dosing of intravenous glofitamab, in 14- or 21-day cycles. Focusing on FL data, an ORR of 66% and a CRR of 57% were observed, with a median DoR that was not reached. CRS occurred in 50% of patients, being G ≥3 in 4%. Only 2 patients (1%) developed G3 ICANS (69). Morschhauser et al. analyzed R/R FL patients treated with the step-up dosing of glofitamab with (n=19) or without (n=21) obinutuzumab, and response rates were similar, with a favorable safety profile regardless of obinutuzumab administration (99).

Epcoritamab is a subcutaneous CD20xCD3 bispecific antibody. Indeed, this route of administration is associated with both a delayed and lower peak of cytokines than the intravenous route, leading to a potentially reduced risk of fatal CRS (70, 97). In the phase 1/2 EPCORE NHL-1 trial, 73 patients with R/R B-NHL received an escalating dose of epcoritamab, and 48 mg was the recommended dose for subsequent trials. Among eleven R/R FL patients, the ORR was 90%, with a CRR of 50%. Adverse events in the entire series included CRS in 59% (all G1-2) and, remarkably, injection site reaction in 47% of patients. Neurological symptoms were reported in 4 patients, all of them transitory.

Data on the 30 FL patients included in the phase 2 EPCORE NHL-2 trial, evaluating the combination of epcoritamab with R (2), were recently presented (100). The ORR was 100%, with a CRR of 93%. At the time of presentation, all had an ongoing response. These impressive outcomes were despite the inclusion of high-risk patients, such as those with primary refractory disease, FLIPI scores 3-5 and POD24. CRS occurred in 50% of patients (G3: 7%) and 1 patient experienced G2 neurotoxicity. Other common treatment-emergent AEs included infection (57%), injection-site reactions (50%), constipation (37%), fatigue (37%) and neutropenia (37%). In a recent update of the study (71, 101), patients in the arm including 66 patients with R/R FL showed an ORR of 95%, with a CRR of 80%. The same combination of epcoritamab and R (2) as frontline treatment was tested in 36 patients with FL, with an ORR of 94% and a CRR of 86%. The toxicity profile was acceptable and comparable between both arms.

Odronextamab is an intravenous CD20xCD3 bispecific engager that was investigated in a phase 1 trial including 145 patients with R/R B-NHL. Forty (28%) patients had the diagnosis of FL and 6 (4%) of MZL. The ORR were 78% and 67% (CR 63 and 33%) for FL and MZL, respectively. In terms of adverse events, CRS was observed in 61% of patients (54% G1-2) and ICANS was found in 12% of patients (G ≥3 in 3%) (72). The pivotal phase 2 study ELM-2 was recently updated: 96 R/R FL patients received odronextamab in 21-day cycles, with an ORR of 81% and a CRR of 75%. These response rates were consistent for the high-risk subgroups, including patients >65 years, POD24, FLIPI 3-5 and patients refractory to their last line of therapy. Most frequently described all-grade AEs were CRS (51%), pyrexia (31%), anemia (31%) and infusion-related reaction (31%) (73).

Other novel bispecific agents targeting CD20, such as plamotamab (102) or imvotamab (IgM-2323) (103) are under investigation, with encouraging preliminary clinical data.

The abovementioned trials evaluate the role of novel agents in heavily pretreated patients who are refractory to ICT, and even in some cases, relapsed after CAR-T therapy. Other ongoing trials are aiming to evaluate the efficacy and safety of these bispecific antibodies as the frontline therapy of FL patients. Examples of these trials include a phase 2 study evaluating the efficacy of mosunetuzumab in combination with polatuzumab vedotin in untreated FL (NCT05410418), and a phase 1b/2 trial of epcoritamab in combination with other standard-of-care agents in subjects with B-NHL (NCT04663347).

### 3.5 Cellular therapy: CAR-T cells

#### 3.5.1 CAR-T cells for follicular lymphoma

In recent years, anti-CD19 chimeric antigen receptor T-cell therapy (CAR-T) has shown significant clinical benefit in phase 3 trials for patients with aggressive B-cell lymphomas, in patients R/R to ≥2 lines of treatment (104–107) and, more recently, in second line in early relapse patients (108–110). In iNHL, the data are still scarce. However, these therapies also seem to have relevant clinical benefit in this setting. Two initial single-institution studies showed that anti-CD19 CAR T-cell therapy might provide clinical benefit for iNHL. First, Schuster et al. of the University of Pennsylvania observed a high rate of response (71% of CR) maintained after 29 mo of follow-up in 14 FL patients who had received a CD19-directed CAR (CTL019). Patients were eligible if they had measurable progression of disease less than 2 years after the second line of ICT. Fifty-seven percent of patients met the criteria for double-refractory FL (progression of disease within 6 months after receiving the last dose of rituximab and the last dose of an alkylating agent) (74). Soon thereafter, Hirayama et al. from Seattle showed similar results in a phase 1/2 trial with 21 patients (8 FL) (76). In both studies treatment was well tolerated. Recently, the long-term outcomes of the trial conducted by the University of Pennsylvania have been reported (111). At 5 years, 43% of patients remained progression-free.

These encouraging data were subsequently confirmed in two trials with two different CD19 CAR-T cell products, axicabtagene ciloleucel (axi-cel) and tisagenlecleucel (tisa-cel) (77, 78). The ZUMA-5 trial (77), the largest phase 2 trial evaluating the use of axi-cel (single intravenous infusion at a target dose of 2×10^6^ CAR-T cells per kg) in R/R indolent lymphomas (FL and MZL) was a single-arm, multicenter trial that granted accelerated FDA approval for the treatment of adults with R/R FL after two or more lines of systemic therapy (112). This trial enrolled 146 patients with histologically confirmed grade 1-3A FL or MZL whose disease had failed to respond to at least 2 prior therapies including an anti-CD20 monoclonal antibody combined with an alkylating agent. More than half of the included patients (55%) were POD24. With a median follow-up of 18 months, axi-cel induced an ORR of 92%, with 76% of patients achieving a CR. The CR ratio was higher in patients with FL (80%) than in those with MZL (60%). At the time of data cutoff, 62% of patients had an ongoing response, and the median DoR, PFS and OS were not reached. Although the study was not powered to assess differences in subgroups, responses were consistent among patients with high-risk disease features, including POD24. A total of 7% and 19% of patients experienced G ≥3 CRS and neurotoxicity, respectively. Of note, a lower proportion of G ≥3 neurological events were reported among patients with FL (15%) than MZL (38%), which was also lower to what has been previously seen in patients with large B-cell lymphoma (32%).

More recently, data from the ELARA trial were reported, a single-arm, multicenter phase 2 trial of tisa-cel in 97 adults with R/R FL (grade 1-3A) following two or more lines of treatment including an anti-CD20 antibody and an alkylating agent, or relapsing after autologous stem cell transplant (78). After lymphodepleting chemotherapy, patients received single-dose tisa-cel (0.6-6×10 (8) CAR viable T cells) on day 1. In 18% of patients, tisa-cel was administrated in the outpatient setting. Interestingly, 63% of included patients were POD24. In the primary analysis of this trial, with a median follow-up of 17 months, a high ORR (86%) and CR (69%) were observed, and the median DoR, PFS and OS were not reached. There was no impact of the dose on best overall response. Toxicities were acceptable and there were no treatment-related deaths. Antitumor activity was seen independently of established risk factors for progression, including heavily pretreated patients, disease refractory to >2 lines of therapy, POD24, bulky disease (64%), advanced disease (86% had stage III-IV disease) or a high-risk FLIPI score (60%). Efficacy in inpatients and outpatients was similar. Dreyling et al. recently presented the long-term clinical outcomes (113). With a follow-up of 29 months, 24 month-PFS, DoR and OS post-infusion was 57%, 65% (78% for patients with CR) and 88%, respectively. An elevated tumor burden [total metabolic tumor volume (TMTV) ≥240 cm (3)] at baseline (pre-LD chemotherapy), POD24, and >4 nodal areas at inclusion were clinical factors correlating significantly with lower efficacy. An assessment of healthcare resource utilization and hospitalization costs was also performed, concluding that outpatients benefitted most (114).

Although differences in patient populations and study designs preclude direct comparisons between trials, FL patients in the ZUMA-5 study had a higher ORR and CR than in ELARA (94% and 79% vs. 86% and 69%, respectively). However, in ZUMA-5, patients with ECOG PS >1 were not included (in the ELARA trial, 43% of patients had an ECOG PS ≥1 before infusion), were less pretreated and did not receive bridging therapy, all of which probably reflects that patients in ZUMA-5 had a higher degree of pre-CAR-T disease control. The timing of initial efficacy assessments for FL patients was comparable in the two studies (1 month). Finally, the safety profile favored tisa-cel. In **Table 2**, the main differences between trials can be seen.

Axi-cel (ZUMA-5) was compared with SCHOLAR-5 as an external control for R/R FL (115). In comparison with available therapies, axi-cel demonstrated substantially improved clinical outcomes (ORR and CRR of 50% and 30% in SCHOLAR-5 vs. 94% and 79% in ZUMA-5), suggesting that CAR-T therapy addresses an important unmet need for R/R FL patients.

#### 3.5.2 CAR-T cells for marginal zone lymphoma

The ZUMA-5 phase 2 trial evaluated the efficacy and safety of axi-cel in 22 MZL patients after at least two lines of therapy (77). Among the 20 MZL patients available for the efficacy analysis, 17 (85%) patients had ORR, with a 55% CR (11 patients) and projected 12-month DoR of 72%. The 12-month PFS and OS rate was 45% and 93%, respectively (77). An updated analysis of efficacy outcomes was performed in patients with MZL by histological categories (nodal vs. extranodal) (77). A higher ORR (100% vs. 76%) and CR (83% vs. 59%) were seen in nodal (n=6) in comparison with extranodal (n=17) MZL. Median time to initial response was 1 month as in FL patients, but the median time to CR was longer than in FL (3 months). The safety profile was manageable, with a similar rate of G ≥3 CRS (8%) but with more frequent G ≥3 neurologic events (38% vs. 15%) than in FL patients (77).

The phase 2, open-label, single-arm, multicenter TRANSCEND FL study (NCT04245839) is ongoing, evaluating lisocabtagene maraleucel (JCAR017) in patients with R/R iNHL (FL and MZL).

### 3.6 Immune checkpoint inhibitors

Several studies recognize the important role of the tumor microenvironment in the pathogenesis of FL (116, 117). The understanding of the crosstalk between neoplastic B-cells and immune cellular components is crucial to design targeted therapies. The impact of PD-1 expression in FL is still controversial (118, 119). The microenvironment of MZL is poorly understood, but some studies are trying to define immune profiles in this lymphoid neoplasm (27).

Anti PD-1 agents such as nivolumab and pembrolizumab, used successfully in other types of lymphoma, have been tested in FL. Although patients with FL treated in the early phase studies of nivolumab showed responses to these agents, with an ORR of 40% and a CRR of 10% (120), these results were not replicated in the phase 2 CheckMate-140 trial. ORR and CR were 4% and 1%, respectively, which makes its use as a single agent somewhat underwhelming (79). Some studies are testing nivolumab in combination with rituximab in treatment-naïve FL patients (NCT03245021), and with lenalidomide in R/R patients (NCT03015896).

Pembrolizumab has also been tested in combination with rituximab in 30 patients with R/R FL. ORR and CR were 67% and 50%, respectively. Median PFS was 13 mo, 3-year OS was 97%, and 23% of patients were still in remission at 35 mo. AEs were tolerable and immune-related AEs were frequently seen, mostly in mild grades. Treatment discontinuation due to AEs occurred in 20% (80). A case report of a SMZL patient who experienced a deep molecular response after receiving pembrolizumab for metastatic melanoma (121) has been published. Pembrolizumab is currently being evaluated in R/R MZL patients in the German Lymphoma Alliance’s POLE-1 trial (NCT03474744).

### 3.7 Magrolimab

The anti-CD47 antibody magrolimab enhances macrophage-mediated phagocytosis binding CD47 and blocking its interaction with SIRPα and cancelling the “don’t eat me signal” (122). In a phase 1 trial of 22 patients with R/R B-NHL (7 FL), magrolimab in combination with rituximab was tested with favorable results (71% of ORR and 43% of CR) and acceptable tolerability (81). Results of the phase 1b/2 part of this study were recently updated, with 35 FL and 2 MZL patients, with an ORR and CR of 66% and 24%, respectively (123). Toxicities included infusion reactions and first dose-related target-anemia, due to splenic phagocytosis of senescent red blood cells. Studies with combinations like venetoclax with obinutuzumab and magrolimab (VENOM) in R/R iNHL are underway (NCT04599634). Other CD47 blockers are also being investigated (124, 125).

## 4 Conclusion

In recent years, many novel therapeutic options with very encouraging results have emerged for patients with indolent lymphomas. Several questions remain unanswered, such as the most effective sequencing of therapies for these patients, the positioning of CAR T-cell therapy in the iNHL treatment algorithm, the most efficient way to preserve immune surveillance function following CAR T-cell therapy to obtain durable results (combination of CAR-T therapy with targeted therapies such as anti-PD1 antibodies as investigated in ZUMA-6, AUTO3 trials for DLBCL, second infusions for patients with antigen-positive relapse), or the role and timing of consolidative hematopoietic stem cell transplantation. Finally, the identification of predictors of relapse after CAR T-cell therapy using methods such as circulating tumor DNA requires investigation in indolent lymphomas.

## Author contributions

All authors contributed to the article and approved the submitted version.
